# The comparison of healthcare utilization inequity between URRBMI and NCMS in rural China

**DOI:** 10.1186/s12939-019-0987-1

**Published:** 2019-06-14

**Authors:** Zengwen Wang, Yucheng Chen, Tianyi Pan, Xiaodi Liu, Hongwei Hu

**Affiliations:** 1 The Center for Social Security Studies of Wuhan University, Wuhan, 430072 China; 2 School of Mathematics and Physics, Anhui University of Technology, Ma Anshan, 243002 China; 3 School of Public Administration and Policy, Renmin University of China, Beijing, 100872 China

**Keywords:** The integration of urban-rural medical insurance, Healthcare utilization, Inequity, Horizontal inequity index

## Abstract

**Background:**

The inequity of healthcare utilization in rural China is serious, and the urban-rural segmentation of the medical insurance system intensifies this problem. To guarantee that the rural population enjoys the same medical insurance benefits, China began to establish Urban and Rural Resident Basic Medical Insurance (URRBMI) nationwide in 2016. Against this backdrop, this paper aims to compare the healthcare utilization inequity between URRBMI and New Cooperative Medical Schemes (NCMS) and to analyze whether the inequity is reduced under URRBMI in rural China.

**Methods:**

Using the data from a national representative survey, the China Health and Retirement Longitudinal Study (CHARLS), which was conducted in 2015, a binary logistic regression model was applied to analyze the influence of income on healthcare utilization, and the decomposition of the concentration index was adopted to compare the Horizontal inequity index (HI index) of healthcare utilization among the individuals insured by URRBMI and NCMS.

**Results:**

There is no statistically significant difference in healthcare utilization between URRBMI and NCMS, but in outpatient utilization, there are significant differences among different income groups in NCMS; high-income groups utilize more outpatient care. The Horizontal inequity indexes (HI indexes) in outpatient utilization for individuals insured by URRBMI and NCMS are 0.024 and 0.012, respectively, indicating a pro-rich inequity. Meanwhile, the HI indexes in inpatient utilization under the two groups are − 0.043 and − 0.028, respectively, meaning a pro-poor inequity. For both the outpatient and inpatient care, the inequity degree of URRBMI is larger than that of NCMS.

**Conclusions:**

This paper shows that inequity still exists in rural areas after the integration of urban-rural medical insurance schemes, and there is still a certain gap between the actual and the expected goal of URRBMI. Specifically, compared to NCMS, the pro-rich inequity in outpatient care and the pro-poor inequity in inpatient care are more serious in URRBMI. More chronic diseases should be covered and moral hazard should be avoided in URRBMI. For the vulnerable groups, special policies such as reducing the deductible and covering these groups with catastrophic medical insurance could be considered.

## Introduction

Amartya Sen once noted that “health is the necessary basis for people to realize other capabilities”. Healthcare service, as the post intervention in diseases, is the last “safety net” to guarantee health, and the equalization of healthcare utilization is an important approach to guarantee health equity [29]. Since the reform and opening up of China, the medical industry and technology have developed rapidly, which also led to an increase in medical expenses. As a result, the inequity in healthcare utilization has also intensified among people of different income groups. Compared to high-income groups, low-income groups have a higher need for healthcare services due to their poor health status [20]. But constrained by income, the healthcare utilization for low-income groups is much less than that of high-income groups.

In China, the inequity in healthcare utilization is more serious in rural areas because there are a large number of low-income groups there [23]. Moreover, for low-income groups, a low level of healthcare utilization can consume most of their wealth, so some of them dare not see a doctor when they are ill. To reduce the inequity in healthcare utilization in rural areas, the Chinese government established the New Cooperative Medical Scheme (NCMS) for rural populations in 2003. By bearing a portion of the medical expenses of the insured, NCMS aims to reduce the economic barriers in healthcare utilization and to improve equity of healthcare services for rural populations regardless of individual socioeconomic factors such as marital status, education and income.

Although the coverage of NCMS has been above 90% since 2008, its services coverage and financial protections are less than those of the Urban Resident Basic Medical Insurance (URBMI) available to the urban populations. According to statistics, “urban residents per capita healthcare expenditure paid by medical insurance is around three times as much as that of the rural residents in 2012”.^1^ The segmentation of the urban-rural medical insurance scheme has become a main factor that caused the urban-rural inequity in healthcare utilization and it is hardly to ensure the healthcare demands of low-income groups in rural areas [24]. This will be harmful to the implementation of rural revitalization strategies and poses a serious threat to social stability in that China is in a critical period of social and economic transformation.

Therefore, the integration of urban-rural medical insurance has gained governmental attention since 2008, and the government established the Urban and Rural Resident Basic Medical Insurance (URRBMI) based on NCMS and URBMI to make residents participate in the same medical insurance regardless of household registration. In 2016, the central government announced that the URRBMI was gradually implemented nationwide in China. Against this backdrop, comparing the inequity in healthcare utilization between URRBMI and NCMS can provide important information for the implementation and development of URRBMI.

Previous studies have revealed that inequity in healthcare utilization exists widely in the world [4, 6, 16]. Apart from inequity between countries, it also exists between regions within one country [1, 3, 21, 25]. The influencing factors of inequity in healthcare utilization are different because of differences in the economy, medical insurance systems and cultures between countries and regions. In previous studies, scholars investigated the relationship between income and the inequity in healthcare utilization, and they found that the inequity is pro-rich in both developed and developing countries, causing an interest transfer between the rich and the poor [4, 6, 17]. Some scholars have also taken other socioeconomic factors into consideration, and they found that factors like medical insurance can also influence the inequity in healthcare utilization [2, 24].

Theoretically, medical insurance can reduce inequity as it provides a financial support in healthcare utilization and covers a large proportion of medical expenses for low-income groups. In China, although medical insurance schemes are a major payment system for healthcare utilization, there are conflicting opinions about the relationship between medical insurance and healthcare utilization inequity. First, some of the studies claim that the implementation of medical insurance narrowed the gap of healthcare utilization [11–13]; second, others argue that medical insurance failed to reduce the degree of inequity in healthcare utilization [17, 18, 26–28, 31], and some scholars also found that medical insurance has intensified the inequity of healthcare utilization instead of narrowing it [2, 23].

The reason that medical insurance fails to reduce healthcare utilization inequity is that the top-level design of the Chinese medical insurance system pays more attention to economic efficiency during the period of primitive accumulation of capital. Studies have shown that the income gap, the fragmentation management of medical insurance and the difference in medical insurance benefits have become the main factors of healthcare utilization inequity [8, 9, 14, 15, 24]. In China, the urban-rural segmentation of the medical insurance system ensures that the rural population experiences lower medical insurance benefits than the urban population. Meanwhile, the proportion of low-income groups in rural areas is higher than that in urban areas. As a result, NCMS cannot meet the healthcare demands of low-income groups and improve the healthcare utilization inequity completely in rural China.

The aim of URRBMI is to provide the same services coverage and financial protections to both urban and rural residents. We assume that the healthcare utilization inequity in rural areas will be reduced as URRBMI improves the medical insurance benefits of rural populations. Referring to URRBMI, some studies found that the opportunity inequity of healthcare utilization was reduced in URRBMI [13], and it also promoted the healthcare utilization of the insured [7, 14, 19, 30]. Meanwhile, other studies implied that the effect of URRBMI on outpatient and inpatient care utilization is not significant [22] and that the rural regional difference in medical insurance benefits still exists under URRBMI [10, 22].

In summary, the previous studies have analyzed the influence of socioeconomic factors on the inequity of healthcare utilization comprehensively, and most of them employed the Concentration Index (CI) to quantify the degree of inequity. Moreover, they also revealed the profound reasons for the healthcare utilization inequity in rural areas by analyzing the urban-rural segmentation of medical insurance, which provided a reference for this article. However, few of them focus on the healthcare utilization inequity in rural areas under URRBMI. Although Ma Chao et al. measured the opportunity inequity of healthcare utilization between urban and rural areas after the integration of urban-rural medical insurance, their research was only limited to three counties in Jiangsu Province of China and did not analyze the inequity within rural areas [13].

Based on the existing studies, this paper analyzes the relationship between URRBMI and the inequity of healthcare utilization in rural areas by comparing the inequity in healthcare utilization under URRBMI and NCMS. Theoretically, the degree of inequity in URRBMI will be lower than in NCMS in that the services coverage and financial protections of URRBMI are better than those of NCMS, but the assumption needs to be verified by data analysis in the next section. This paper is organized as follows:

First, as the fact that healthcare utilization is mainly affected by health status and socioeconomic factors, we use the chi-square test to make a comparison of differences in healthcare utilization and other influence factors among the individuals who were insured by URRBMI and NCMS.

Second, as the dependent variables in this paper are discrete, and the inequity in healthcare utilization is income-related, the binary logistic regression model is employed in this paper to analyze the differences in healthcare utilization among different income levels under the two medical insurance schemes.

Third, the concentration index (CI) and horizontal inequity index (HI index) are used to quantify the degree of inequity in healthcare utilization under the two groups, the decomposition of the concentration index is used to present the contribution of each independent variable to the concentration index.

## Methods

### Data source

The data analyzed in this paper are from the China Health and Retirement Longitudinal Study (CHARLS) in 2015.^2^ This is a high-quality micro database about individuals who are aged 45 and above in China, and the database was established by the National Development Research Institute of Peking University. These data cover around 12,400 households and 23,000 respondents in different regions of China by using the method of population proportion sampling (PPS), and 450 communities of 150 counties in 28 provinces were interviewed in this investigation. Therefore, these data have a good sample representation. The data questionnaire contains information about individuals’ demographics, health status, healthcare utilization, medical insurance, family structure and income. According to the household registration status, there are 12,426 rural respondents in the 2015 CHARLS data, and the respondents studied in this paper are individuals who were insured only by URRBMI or NCMS. After removing the study subjects with missing variables data, those who changed the household registration (having a rural household registration but participating in URBMI) and whose per capita household income was less than 0, the final effective sample size was 9002. All respondents were weighted by sampling probability.

### Measurement of healthcare utilization, health needs and socioeconomic factors

In general, healthcare utilization includes outpatient and inpatient care. Based on the characteristics of the selected data, this paper selects “outpatient visit in the last month” and “inpatient visit in the last year” as indicators to measure healthcare utilization. In addition, referring to previous studies, health need factors include gender, self-assessed health and chronic morbidity; socioeconomic factors include education level, per capita household income, living areas and community. The detailed definition of variables can be seen in Table 1.

**Table 1:** The definition of variables

Variable	Definition
Healthcare utilization	
One-month outpatient visit	Visited a doctor in the last month; Yes = 1; No = 0
Inpatient visit	Received inpatient care in the last year; Yes = 1; No = 0
New cooperative medical insurance (NCMS)	Insured by new cooperative medical insurance; Yes = 1; No = 0
Urban and rural resident basic medical insurance (URRBMI)	Insured by urban and rural resident basic medical insurance; Yes = 1; No = 0
Health needs factors	
Gender	Male = 1; Female = 0
Age	If 45 = <age < 60, age = 0; If age > =60, age = 1
Self-reported health	Excellent = 1; Very good = 2; Good = 3; Fair = 4; Poor = 5
Chronic	At least has one chronic; Yes = 1; No = 0
Socioeconomic factors	
Marital status	Married/cohabiting = 1; Single/divorced/widowed = 0
Educational level	Illiterate = 0; Primary = 1; Senior/middle = 2 College or high = 3
Average household income	The average household incomes per capita
Region	Eastern = 0; Central = 1; Western = 2
Community	The respondents’ address is in town center or combination zone between urban and rural or city zone = 1; Others = 0

### Statistical analysis

First, this paper uses the chi-square test to examine whether there are significant differences in healthcare utilization and other influence factors among individuals insured by URRBMI and NCMS.

### Binary logistic regression

Since the dependent variables are discrete and the inequity in healthcare utilization is income-related, we employed binary logistic regression to analyze the differences in healthcare utilization among different income levels under the two medical insurance schemes. The marginal effect estimated in this model will be used to decompose the concentration index in the next part. The model is as follows:

1 $$ \ln \left(\frac{p}{1-p}\right)=\alpha +{\sum}_j{\beta}_j^m{x}_{ji}+{\sum}_k{\gamma}_k^n{x}_{ki}+{\varepsilon}_i $$

p/(1-p) indicates the healthcare utilization; *x*_*ji*_ indicates health needs variables; *x*_*ki*_ indicates the socioeconomic variables; ${\beta}_j^m$ and ${\gamma}_k^n$ indicate the marginal effects of each variable; and *ε*_*i*_ indicates the error term. The income variable in *x*_*ki*_ is the focus in this model.

### Measurement of healthcare utilization inequity

The Concentration Index is employed to quantify the inequity degree of healthcare utilization relating to socioeconomic characteristics, reflecting the changes in the distribution of healthcare utilization among different socioeconomic groups [4]. The Concentration Index can be given by the following equation:

2 $$ \mathrm{C}=2/\upmu \mathrm{cov}\left({y}_i,{r}_i\right) $$

C is the Concentration Index; *y*_*i*_ represents the status of healthcare utilization of individual *i*; *r*_*i*_ indicates the scoring rank of income of individual *i*; and *μ* is the mean of healthcare utilization. The Concentration Index is defined as twice the area between the concentration curve and the line of equality.^3^ In the graph of the concentration curve, the diagonal is the line of equality. The value of the Concentration Index ranges from − 1 to 1. In addition, if the concentration curve coincides with the diagonal, the value of the Concentration Index is 0, meaning that there is no healthcare utilization inequity; if the concentration curve is above the diagonal, meaning the value of the Concentration Index is negative, there is pro-poor inequity in healthcare utilization; if the concentration curve is below the diagonal, meaning the value of the Concentration Index is positive, then the healthcare utilization is concentrated among the rich.

The concentration index indicates the overall inequity in healthcare utilization. However, healthcare utilization is influenced by a variety of factors, including health needs and socioeconomic factors. The World Health Organization defines the equalization of healthcare utilization as “People who have similar health needs should have the same healthcare utilization”, which indicates that differences in healthcare utilization caused by different health status is reasonable. Therefore, this paper employs the horizontal inequity index to quantify the healthcare utilization inequity caused by the differences in socioeconomic factors, rather than the differences in health needs. By decomposing the concentration index, the contribution of health needs and socioeconomic factors to the inequity of healthcare utilization can be measured separately. The horizontal inequity index can be obtained by subtracting the contribution of health needs from the total concentration index, and the absolute value of it measures the degree of inequity. The specific definitions of variables are shown in Table 1.

The decomposition formula of the concentration index is:

3 $$ C={\sum}_j\left({\beta}_j^m\overline{x_j}/\overline{y}\right){C}_j+{\sum}_k\left({\gamma}_k^n\overline{z_k}/\overline{y}\right){C}_k+G{C}_u/\overline{y} $$

C is the total concentration index; $\overline{y}$ is the mean of healthcare utilization (including outpatient care and inpatient care), $\overline{x_j}$ and $\overline{z_k}$ are the mean values of *x*_*j*_ and *z*_*k*_, respectively; C_j_ and C_k_ are the concentration index of the health needs and of the socioeconomic variables, respectively; and GC_u_ is the concentration index of the error term. The first part of the formula represents the contribution of health needs to inequity, and the second is the contribution of socioeconomic factors.

## Results

### Descriptive analysis

Table 2 shows the results of the Chi-square test. As shown in the table, 3593 individuals are insured by URRBMI and 5409 are insured by NCMS.^4^ The differences in healthcare utilization between the two groups are not significant, and the individuals who are insured by NCMS show greater health needs, but the differences are also not significant. However, there are significant socioeconomic differences (education, per capita household income and region) between the URRBMI insured and NCMS insured. Specifically, the socioeconomic status of the population insured by URRBMI is better than that of the population insured by NCMS, especially in per capita household income.

**Table 2:** Summary statistics of variables in URRBMI and NCMS

	URRBMI	NCMS	χ^2^	*P* value
Healthcare utilization				
One-month outpatient visit	734 (20.43)	1072 (19.82)	0.501	0.479
inpatient visit	471 (13.11)	687 (12.70)	0.320	0.571
Health needs				
Gender			0.489	0.485
Male	1720 (47.87)	2630 (48.62)		
Female	1873 (52.13)	2779 (51.38)		
Age			0.0716	0.789
45–60	1764 (49.10)	2640 (48.81)		
60+	1829 (50.90)	2769 (51.19)		
Self-assessed Health			4.996	0.288
Excellent	38 (1.06)	60 (1.11)		
Very Good	385 (10.72)	555 (10.26)		
Good	396 (11.02)	599 (11.07)		
Fair	1955 (54.41)	2856 (52.80)		
Poor	819 (22.79)	1339 (24.76)		
Chronic			2.550	0.110
Sick	2738 (76.20)	4200 (77.65)		
Not Sick	855 (23.80)	1209 (22.35)		
Socioeconomic factors				
Marital Status			0.228	0.633
Married/cohabiting	3164 (88.06)	4745 (87.72)		
Single/divorced/widowed	429 (11.94)	664 (12.28)		
Education			8.563	0.036
illiterate	844 (23.49)	1416 (26.18)		
Primary	1308 (36.40)	1925 (35.59)		
Junior/Senior High School	718 (19.98)	1028 (19.01)		
College or Higher	723 (20.12)	1040 (19.23)		
Per capita Household Income			72.514	0.000
The Lowest	697 (19.40)	1103 (20.39)		
The Second	603 (16.78)	1156 (21.37)		
The Third	674 (18.76)	1166 (21.56)		
The Fourth	778 (21.65)	1000 (18.49)		
The Highest	841 (23.41)	984 (18.19)		
Region			25.109	0.000
East	1283 (35.71)	1703 (31.48)		
Central	1184 (32.95)	1762 (32.58)		
West	1126 (31.34)	1944 (35.94)		
Community			0.096	0.757
Town central, city zone or zone between them	184 (5.12)	285 (5.27)		
Zhenxiang area, special area, township central or village	3409 (94.88)	5124 (94.73)		
Sample size	3593	5409		

The per capita of household income is divided into five quintiles, 0 ≤ the lowest ≤17; 17 < the second ≤495; 495 < the third ≤2587; 2587 < the fourth ≤9992; and the highest > 9992, unit (Yuan)

### Binary logistic regression analysis

Before measuring the inequity of healthcare utilization, we used binary logistic regression to explore the influence of income on healthcare utilization. Table 3 and Table 4 present the logistic regression results on outpatient and inpatient care, respectively. In terms of outpatient care, there are no statistical disparities among the different income levels in URRBMI. However, among the individuals who are insured by NCMS, the high-income groups tend to have significantly higher healthcare utilization. With regard to inpatient care, as Table 4 shows, there are no significant disparities among different income groups, regardless of the medical insurance schemes. Besides, compared to the highest income groups, the lowest groups show greater utilization of inpatient care, although it is not significant. For other control variables, the health status of individuals who are insured by both insurance schemes has a significant influence on healthcare utilization. The marginal effect estimated in this part was used for the decomposition of the concentration index, and all values were weighted by the sampling probability.

**Table 3:** Binary logistic regression on one-month outpatient utilization

One-month outpatient visit				
Variables	URRBMI (dy/dx)	95% CI	NCMS (dy/dx)	95% CI
Per capita Household Income (Ref: the lowest)				
The second	− 0.004	[− 0.049, 0.040]	0.030	[− 0.007, 0.068]
The third	−0.010	[− 0.053, 0.033]	0.044^**^	[0.005, 0.083]
The fourth	− 0.005	[− 0.047, 0.037]	0.001	[− 0.035, 0.038]
The highest	0.023	[−0.022, 0.067]	0.046^**^	[0.004, 0.087]
Gender (Ref: female)	−0.036^**^	[− 0.065, − 0.008]	− 0.053^***^	[− 0.078, − 0.028]
Age (Ref: 45–60)	− 0.013	[− 0.045, 0.018]	0.017	[− 0.008, 0.042]
Self-assessed health (Ref: poor)				
Excellent	− 0.062	[− 0.156, 0.033]	− 0.130^***^	[− 0.184, − 0.076]
Very good	− 0.137^***^	[− 0.168, − 0.107]	− 0.150^***^	[− 0.172, − 0.128]
Good	− 0.119^***^	[− 0.151, − 0.087]	−0.122^***^	[− 0.148, − 0.096]
Fair	−0.099^***^	[− 0.131, − 0.067]	−0.109^***^	[− 0.134, − 0.084]
Chronic (Ref: not sick)	0.090^***^	[0.060, 0.120]	0.075^***^	[0.047, 0.104]
Marital Status (Ref: single/divorced/widowed)	−0.007	[− 0.051, 0.036]	0.000	[− 0.034, 0.035]
Education (Ref: illiterate)				
Primary	0.014	[− 0.022, 0.051]	0.001	[−0.029, 0.031]
Junior/senior high school	0.004	[− 0.043, 0.050]	0.040^*^	[− 0.002, 0.082]
College or higher	0.011	[−0.036, 0.058]	0.002	[−0.037, 0.040]
Region (Ref: east)				
Central	−0.006	[− 0.039, 0.027]	0.044^***^	[0.012, 0.077]
West	0.022	[−0.013, 0.057]	0.063^***^	[0.032, 0.094]
Community (Ref: township and village)	0.029	[−0.036, 0.093]	−0.036	[− 0.094, 0.021]
Sample size	3593		5409	

The dy/dx in brackets indicates the marginal effect; * *p* < 0.10; ** *p* < 0.05; *** *p* < 0.01; The CI is confidence interval

**Table 4:** Binary logistic regression on inpatient utilization

Inpatient visit				
Variables	URRBMI (dy/dx)	95% CI	NCMS (dy/dx)	95% CI
Per capita Household Income (Ref: the lowest)				
The second	0.024	[−0.016, 0.064]	−0.009	[− 0.035, 0.017]
The third	0.008	[−0.027, 0.044]	0.003	[−0.024, 0.030]
The fourth	0.006	[−0.030, 0.042]	−0.002	[− 0.029, 0.024]
The highest	−0.005	[− 0.042, 0.031]	−0.020	[− 0.048, 0.007]
Gender (Ref: female)	0.017	[−0.007, 0.041]	0.000	[−0.019, 0.018]
Age (Ref: 45–60)	0.029^**^	[0.006, 0.053]	0.017^*^	[−0.002, 0.036]
Self-assessed health (Ref: poor)				
Excellent	/	/	−0.070^***^	[−0.112, − 0.029]
Very good	− 0.106^***^	[− 0.130, − 0.082]	−0.094^***^	[− 0.111, − 0.078]
Good	− 0.078^***^	[− 0.101, − 0.055]	−0.076^***^	[− 0.094, − 0.057]
Fair	−0.093^***^	[− 0.119, − 0.066]	− 0.101^***^	[− 0.121, − 0.081]
Chronic (Ref: not sick)	0.058^***^	[0.029, 0.087]	0.063^***^	[0.042, 0.084]
Marital Status (Ref: single/divorced/widowed)	−0.073^***^	[− 0.118, − 0.027]	−0.024^*^	[− 0.052, 0.004]
Education (Ref: illiterate)				
Primary	−0.001	[−0.030, 0.028]	− 0.011	[− 0.032, 0.011]
Junior/senior high school	− 0.003	[− 0.040, 0.034]	−0.010	[− 0.037, 0.017]
College or higher	−0.025	[− 0.059, 0.009]	−0.004	[− 0.033, 0.025]
Region (Ref: east)				
Central	−0.004	[− 0.032, 0.024]	0.007	[−0.017, 0.030]
West	0.038^**^	[0.006, 0.069]	0.023^*^	[−0.001, 0.046]
Community (Ref: township and village)	0.004	[−0.047, 0.054]	−0.022	[− 0.058, 0.014]
Sample size	3555		5409	

The dy/dx in brackets indicates the marginal effect; * *p* < 0.10; ** *p* < 0.05; *** *p* < 0.01; The CI is confidence interval. The population that report their health status as excellent in the URRBMI group has not received inpatient care in the past year, so the excellent row has no data

### Measurement of inequity of healthcare utilization

Table 5 shows the concentration index of healthcare utilization among the individuals insured by the two medical insurance schemes. The concentration index of outpatient care in URRBMI is positive but small, indicating a tendency of pro-rich inequity in outpatient care, and the concentration index of inpatient care in URRBMI is negative, indicating a pro-poor inequity in inpatient care. In NCMS, both outpatient and inpatient care are negative, which means that they are concentrated in the poor. However, the values in Table 5 represent the concentration index of healthcare utilization that is influenced by health needs and socioeconomic factors, which not fully reflect the inequity. We then make a decomposition of the concentration index in the next part.

**Table 5:** Concentration index (CI) of healthcare utilization

	URRBMI (CI)	NCMS (CI)
One-month outpatient visit	0.005	−0.012
Inpatient visit	−0.083	−0.053

All values are weighted

Table 3 and Table 4 have shown the marginal effect of the independent variables that is estimated from the binary logistic regression, which is used to decompose the concentration index of healthcare utilization in Eq. 3.

Table 6 and Table 7 show the contributions of independent variables on the concentration index of healthcare utilization in URRBMI and NCMS, and the percentage of each variable’s contribution to the concentration index is also included. From Table 6, we can see that per capita household income shows the greatest contribution to the concentration index of outpatient care in URRBMI and NCMS (401.004% and − 199.016%, respectively), and the second largest is self-assessed health (− 243.945 and 95.848%, respectively). With regard to inpatient care, the contribution percentage of per capita household income to the concentration index is also the greatest in URRBMI and NCMS (22.228 and 39.707%, respectively).

**Table 6:** The contribution of each independent variable to the inequity in one-month outpatient utilization

One-month outpatient visit				
Variables	URRBMI		NCMS	
	Contribution	%	Contribution	%
Health needs				
Gender (Ref: female)	−0.002	−33.767	− 0.003	22.213
Age (Ref: 45–60)	0.005	90.462	−0.004	33.257
Self-assessed health (Ref: poor)		−243.945		95.848
Excellent	0.000	−5.283	−0.002	12.791
Very good	−0.006	−117.140	− 0.006	50.428
Good	−0.003	−56.059	−0.002	17.676
Fair	−0.004	−65.463	− 0.002	14.953
Chronic (Ref: not sick)	−0.009	−166.180	− 0.005	43.070
Socioeconomic factors				
Marital status (Ref: single/divorced/widowed)	−0.001	−11.915	0.000	−0.100
Education (Ref: illiterate)		21.991		20.638
Primary	0.000	−5.643	0.000	0.463
Junior/senior high school	0.000	6.974	0.002	−19.571
College or higher	0.001	20.660	0.000	−1.530
Per capita Household Income (Ref: the lowest)		401.004		−199.016
The second	0.002	28.469	−0.013	102.835
The third	0.001	16.048	0.000	2.463
The fourth	−0.002	−27.867	0.001	−4.874
The highest	0.021	384.354	0.037	− 299.440
Region (Ref: east)		−9.933		35.751
Central	0.000	6.274	−0.005	37.437
West	−0.001	−16.207	0.000	−1.686
Community (Ref: township and village)	0.002	44.027	−0.003	22.846

All values are weighted by sampling probability

**Table 7:** The contribution of each independent variable to the inequity in inpatient utilization

Inpatient visit				
Variables	URRBMI		NCMS	
	Contribution	%	Contribution	%
Health needs				
Gender (Ref: female)	0.001	−1.575	0.000	0.038
Age (Ref: 45–60)	−0.016	19.239	− 0.006	11.975
Self-assessed health (Ref: poor)		19.062		22.525
Excellent	/	/	−0.001	2.453
Very good	−0.008	9.554	−0.006	11.249
Good	−0.003	3.312	−0.002	3.895
Fair	−0.005	6.196	−0.003	4.928
Chronic (Ref: not sick)	−0.009	10.538	−0.007	12.821
Socioeconomic factors				
Marital status (Ref: single/divorced/widowed)	−0.009	11.442	−0.002	3.514
Education (Ref: illiterate)		5.086		1.544
Primary	0.000	−0.027	0.001	−1.534
Junior/senior high school	−0.001	0.613	−0.001	1.751
College or higher	−0.004	4.500	−0.001	1.327
Per capita Household Income (Ref: the lowest)		22.228		39.707
The second	−0.013	15.591	0.006	−10.441
The third	−0.001	1.312	0.000	0.058
The fourth	0.003	−3.010	−0.002	2.870
The highest	−0.007	8.335	−0.025	47.220
Region (Ref: east)		2.09		1.824
Central	0.000	−0.433	−0.001	2.042
West	−0.002	2.523	0.000	−0.218
Community (Ref: township and village)	0.000	−0.538	−0.003	4.812

All values are weighted by sampling probability. The population that reports their health status as excellent in the URRBMI group has not received inpatient care in the past year, so the excellent row has no data

The horizontal inequity index can be obtained by subtracting the contributions of health needs variables from the concentration index in healthcare utilization. Table 8 shows that the horizontal inequity indexes of outpatient utilization for the URRBMI insured and NCMS insured are 0.024 and 0.012, indicating that there is a pro-rich inequity in outpatient utilization. In other words, with the same need for outpatient care, the rich people utilize more outpatient care than the poor people in both the URRBMI and NCMS groups, and the inequity degree of outpatient utilization in URRBMI is larger than that in NCMS. Differing from outpatient care, with the reported horizontal inequity indexes of − 0.043 and − 0.028, the inequity of inpatient utilization for the URRBMI insured and NCMS insured is pro-poor, indicating that the poor utilize more inpatient care in the two groups. This seems to reduce the inequity that described in the introduction because of the poor’s greater need for healthcare. However, on one hand, the inequity that measured by the horizontal inequity index has been standardized for differences in need and it is only related to socioeconomic factors. On the other hand, excessive utilize healthcare services not only damages health, but also wastes medical resources. Especially in the case of medical insurance sharing a large proportion of hospitalization expenses, the poor are more sensitive to the price of healthcare than the rich, and the pro-poor inequity here can be regarded as the moral hazard of medical insurance. Similarly, the absolute value of the horizontal inequity index of inpatient utilization for the URRBMI insured is also larger than that of the NCMS insured, indicating a larger inpatient utilization inequity for the URRBMI insured.

**Table 8:** Horizontal inequity index of healthcare utilization among the two medical insurance schemes

	One-month outpatient visit		Inpatient visit	
	URRBMI	NCMS	URRBMI	NCMS
CI	0.005	−0.012	− 0.083	−0.053
Contributions of health needs	−0.019	−0.024	− 0.04	−0.025
HI index	0.024	0.012	−0.043	−0.028

All values are weighted

## Discussion

The urban-rural segmentation of medical insurance schemes has become an important factor of healthcare utilization inequity in rural China. To provide better coverage services and financial protection for the rural population and to reduce the healthcare utilization inequity in rural areas, China began to establish Urban and Rural Resident Basic Medical Insurance (URRBMI) based on Urban Resident Basic Medical Insurance (URBMI) and the New Cooperative Medical Scheme (NCMS) nationwide in 2016. Against this backdrop, it is worth analyzing the changes in inequity in healthcare utilization after the implementation of URRBMI.

Using the national representative data from the China Health and Retirement Longitudinal Study (CHARLS) that was conducted in 2015, this paper compares the inequity of healthcare utilization between the URRBMI insured and the NCMS insured in rural China. The results reveal that there is no significant difference in healthcare utilization under the two medical insurance schemes. When comparing the inequity under the two medical insurance schemes, we find that the richest utilize more outpatient care in URRBMI, but the difference is not significant. Besides, in the NCMS group, the high-income group tends to utilize significantly more outpatient care. The horizontal inequity index (HI index) also shows that there is a pro-rich inequity of outpatient care in both the URRBMI and NCMS groups and that the HI index of URRBMI is larger than that of NCMS; the result is similar to Chen’s study, which also found that the individuals insured by NCMS have a pro-rich inequity in outpatient utilization [3]. The reason may be related to the characteristics of medical insurance policy in China. As the main aim of NCMS is to reduce the financial barriers in hospitalization and the critical diseases of outpatients, and the URRBMI in rural China was established on the basis of NCMS, so its reimbursement policy also places a higher emphasis on inpatient care initially. The individuals who are insured by URRBMI and NCMS in rural China need to pay most of their outpatient fees. Besides, the high-level hospitals mean better quality of healthcare, so people are more inclined to seek healthcare in high-level hospitals if it is possible. As a result, the individuals who are insured by URRBMI tend to seek outpatient care in high-level hospitals because they think the URRBMI has a higher benefit, which leads to higher medical expenses and a serious pro-rich inequity [5]. For the individuals who are insured by NCMS, most of them seek outpatient care in village clinics. Although the expenses of outpatient care are lower overall, some low-income populations still cannot afford of them.

In terms of inpatient utilization, binary logistic regression shows no significant difference among different income levels in both URRBMI and NCMS. However, the horizontal inequity index (HI index) reveals the inequity behind it, that pro-poor inequity exists in inpatient utilization under the two medial insurance schemes, indicating that the poor utilize more inpatient care than the rich. The absolute value of the HI index in URRBMI is larger than the absolute value in NCMS, meaning that the degree of inequity of URRBMI is higher than that of NCMS. Additionally, the degree of inequity in inpatient is higher than that in outpatient care. The result on inequity of inpatient care among those insured by NCMS is different than those reported for other studies, such as Chen (2018) and Pan (2017), who both found a pro-rich inequity in inpatient care in NCMS [3, 18]. The reason for the pro-poor inequity in our results may be that the NCMS has a high reimbursement rate on inpatient utilization. A large proportion of inpatient expenses can be reimbursed, indicating that the insured face a lower price of inpatient services. In this case, the low-income groups will utilize more inpatient care because they are more sensitive to changes in prices than the high-income groups. In addition, as the aim of URRBMI is to increase the rural residents’ medical insurance benefits and to reduce the disparities between urban and rural areas, the central and local governments have arranged lots of resources to finance the URRBMI. Thus, URRBMI has a higher benefit level than NCMS, such as a higher reimbursement rate in inpatient care [7], that caused a greater pro-poor inequity.

It is also important to bear in mind that there is limitation in this paper. Since the implementation of URRBMI is not a ‘natural experiment’, it is hardly to avoid the problem of self-selection in this process. Therefore, the results of this paper are statistical analysis rather than ‘causal’ analysis. Although the PSM method can solve the self-selection problem to some extent by matching the treatment and control groups, it cannot control the unobservable characteristics (such as the willingness of local leaders). Besides, the PSM has several candidate matching methods, such as one-to-one, one-to-four, radius and kernel matching et al. If the PSM method was applied, we must use the matched data to calculate the concentration index of healthcare utilization among the treatment and control groups, respectively. Against this backdrop, only the one-to-one method can be applied. However, the results we get will not be accurate because large numbers of samples are deleted, and it is also difficult to test the robustness of the PSM matching effect only by the one-to-one matching. As a result, the ‘causal’ relationship cannot be attained in this paper. Meanwhile, there are few studies to analyze the inequity of healthcare utilization in URRBMI and to compare it with that in NCMS. It is reasonable to make a statistical analysis firstly, and the future study could further identify the ‘causal’ effect between the inequity of healthcare utilization and URRBMI.

## Conclusion

This paper shows that inequity still exists in rural areas after the integration of urban-rural medical insurance schemes, and there is a certain gap between the actual and the expected goal of URRBMI. Specifically, compared to NCMS, the pro-rich inequity in outpatient care and the pro-poor inequity in inpatient care are more serious in URRBMI. Thus, comprehensive measures to reduce the inequity should be taken: first, covering more outpatient diseases in URRBMI, especially chronic diseases; second, setting a reasonable reimbursement ratio in inpatient care to avoid the moral hazard in URRBMI; and third, for the vulnerable groups, special policies such as reducing the deductible and covering them in catastrophic medical insurance could be considered.

## Data Availability

The datasets used during the current study are not publicly available due to the confidential policy but are available by applying on the CHARLS official website.

## Abbreviations

CHARLS: China Health and Retirement Longitudinal Study
CI: Concentration Index
HI: Horizontal inequity
NCMS: New Cooperative Medical Insurance
PPS: Population proportion sampling
URBMI: Urban Residents Basic Medical Insurance
URRBMI: Urban and Rural Residents Basic Medical Insurance

## Footnotes

1 The data comes from China Statistical Yearbook.

2 Although the Chinese government began to promote the integration of urban-rural medical insurance nationwide in 2016, some provinces have piloted it previously. By 2014, seven provinces such as Tianjin, Qinghai, Ningxia, Guangdong, etc., had completed the integration of urban-rural medical insurance.

3 The concentration curve was first put forward by Wagstaff, and it is used to measure the disparities in health distribution that are caused by different socioeconomic characteristics.

4 In CHARLS 2015, there are only hundreds of rural samples with URRBMI in that some rural respondents do not know that they have covered by URRBMI. Many respondents who have been covered by URRBMI think that they are still covered by NCMS. Besides, URRBMI is implemented at the county level, and the CHARLS provides the respondent’s provincial, city and community information. Therefore, when a community has respondents who are insured by URRBMI, we treat all respondents of the community as being covered by URRBMI.
